# Comparison of Factors Influencing Gestational Outcomes in Healthy Versus Hypothyroid Women from Karachi, Pakistan

**DOI:** 10.34172/aim.28564

**Published:** 2024-08-01

**Authors:** Zareen Kiran, Adeel Khoja, Imdad Ali Khushk, Aisha Sheikh, Najmul Islam

**Affiliations:** ^1^Department of Medicine, Aga Khan University Hospital, Karachi, Pakistan; ^2^Health Services Academy, Islamabad, Pakistan

**Keywords:** Gestation, Outcome, Overt, Risk factor, Sub-clinical, Thyroid

## Abstract

**Background:**

Gestational outcomes are known to be negatively correlated with hypothyroidism. This study was designed to compare the maternal factors affecting gestational outcomes in women with and without hypothyroidism.

**Methods:**

This retrospective analysis was carried out in a tertiary hospital in Karachi, Pakistan, between 2008 and 2016. A standardized form was used to collect information on the age of the mother, gestational duration at the prenatal appointment, gestational diabetes mellitus (GDM), hypertension, and past records of miscarriages in hypothyroid and healthy pregnant women. Gestational outcomes were recorded as live birth or pregnancy loss. Statistical analysis was performed to examine overt versus sub-clinical hypothyroidism and among those diagnosed before versus during gestation.

**Results:**

A collective of 708 women were enlisted in the hypothyroid pregnant group and 759 were recruited in healthy controls. Pregnancy loss was 9.9% (n=70) in hypothyroid women, whereas it was 14.3% (n=108) in the control group. The age of the mother, gestational duration at the prenatal appointment, and past records of miscarriages were discovered to be related to a higher chance of pregnancy loss in a multivariable analysis, but GDM (OR 0.04, CI 0.06-0.32, *P*=0.002) and hypothyroidism (OR 0.62, CI 0.43-0.89, *P*=0.01) exhibited a protective effect.

**Conclusion:**

This study found the age of the mother, gestational duration at a prenatal appointment, and past records of miscarriages to be associated with negative outcomes in hypothyroidism. These factors remained significant in overt as well as subclinical hypothyroid women.

## Introduction

 Hypothyroidism is predicted to occur in 4% of pregnancies worldwide, including 0.5% with overt hypothyroidism and 3.5% with subclinical hypothyroidism.^1^ The prevalence of hypothyroidism during gestation has been reported to be 1-1.6% in a multi-center study from Pakistan.^2^ Complications like gestational hypertension (GH)^3^ and miscarriages^4^ are observed to be related with hypothyroidism in pregnancy. Preterm birth,^5^ placental abruption, and stillbirth^6^ have also been linked to hypothyroidism. Both autoimmune thyroid disease- and non-autoimmune thyroid disease-affected euthyroid women experience an impaired thyroid milieu during gestation and a significant increase in obstetric problems.^7,8^ According to an Indian study, women with overt hypothyroidism were observed to have a higher likelihood of experiencing GH, intrauterine growth restriction (IUGR), and intrauterine fetal demise.^9^ A Pakistani investigation on patients with gestational diabetes mellitus (GDM) found that 61.5% had subclinical hypothyroidism.^10^

 Obstetric outcomes have been described in several studies for both overt and subclinical hypothyroidism; however, only few have addressed the influence of factors on negative gestational outcomes in this population.^6,11^ The main reason for this is lack of consistency among population-based research in which thyroid hormone axis is influenced by nutritional iodization of the population, effect of environmental and genetic factors, and the incidence of autoimmune phenomena in the population. For example, in a low risk population, the age of the mother, blood pressure status or maternal smoking status did not show any difference between normal and subclinical hypothyroid groups.^12^ Another study reported no effect of thyroid antibody status on abortions; however, there was a significant effect of maternal parity on GH, and the effect of the coexistence of impaired glucose homoeostasis (GDM) and chronic hypertension on postpartum hemorrhage in antibody positive women.^13^

 Overall, South Asian studies on factors affecting gestational outcomes in women with hypothyroidism are scarce. Therefore, the objective of this study was to investigate the factors that influence the gestational outcomes of women with hypothyroidism, whether identified preconception or during the antenatal period, and in both overt and subclinical hypothyroid groups.

## Materials and Methods

###  Data Collection

 We collected data on pregnant women who visited either endocrine or maternity clinics at AKUH between 2008 and 2016. Patients were allocated into a hypothyroid group, and a control group without any medical diseases. At their first visit, the data on the age of the mother and gestational duration (trimesters) was recorded. Comorbid variables in the hypothyroid group included a history of diabetes mellitus before conception, GDM, and hypertension (chronic hypertension, GH, and preeclampsia). In both groups, we identified past records of miscarriages. We divided gestational outcomes into live births and pregnancy loss.

###  Patient Selection

 Pregnant women visiting endocrine or maternity clinics and diagnosed with hypothyroidism by their doctor were included in the hypothyroid group. The healthy pregnant group consisted of women with no comorbidities. The study excluded pregnant women with thyroid hormone test results within the normal range.

###  Sample Size

 The gestational outcomes of hypothyroid-affected pregnancies revealed that spontaneous abortions were 15.4% in women with subclinical hypothyroidism.^14^ We calculated the sample size assuming a prevalence of 16% with a 95% confidence interval, and a 5% margin of error (alpha error) to recruit 278 pregnant women for this specific study. This sample size calculation is powered at 80% with a 10% non-response rate.

###  Gestational Outcomes

 Gestational outcomes were described as either live birth or pregnancy loss. Pregnancy loss can mean abortion and intrauterine death (IUD), or stillbirth; defined as per international criteria.^15^ Medical termination of pregnancy as defined by the international clinical guidelines may also be included.^16^ Ectopic pregnancy, as described clinically and radiologically, was also included.^17^

###  Statistical Analysis

####  Descriptive Statistics

 For all data analyses, Stata version 12 was employed. The hypothyroid and control groups’ independent variables were expressed as mean with SD for normally distributed continuous data based on histogram (graphical representation) and Shapiro-Wilk test (computing technique) and median with interquartile range (IQR) for skewed distributed data. Frequencies and percentages presented categorical variables.

####  Inferential Statistics

 In this study, the binary logistic regression analysis was employed to assess the relationship between independent factors and gestational outcomes. The linearity assumption underlying logistic regression analysis for quantitative variables was assessed. In the univariate regression model, *P *value was taken as < 0.25 to create a parsimonious regression model. All significant variables were analyzed in multivariable logistic regression analysis, following a stepwise selection model building approach, using chi square taking the *P *value cut-off at ≤ 0.05. When the expected cell count was < 5, we adopted the Fisher’s exact test to calculate the significance of the association. For the manual model building approach, a *P *value of < 0.25 was used as the entry criterion and variables having a *P *value > 0.05 were removed from the model in order to obtain a parsimonious multivariable model.

## Results

 The average age among hypothyroid pregnant women was 30.91 years ( ± 5.68), whereas it was 29.44 years ( ± 4.85) in healthy pregnant women. Table 1 classifies independent factors into two groups: hypothyroid pregnant women (n = 708) and healthy pregnant women without comorbidities (n = 759).

**Table 1:** Independent Maternal Factors Among the Pregnant Population

**Maternal Factors**	**Pregnant Women with Hypothyroidism (n=708)** **No. (%)**	**Healthy Pregnant Women (n=759)** **No. (%)**
Gestational outcomes		
Live birth	638 (90.1%)	650 (85.7%)
Pregnancy loss	70 (9.9%)	108 (14.3%)
Age of the mother		
19 to 30 years	340 (48.0%)	520 (68.5%)
31 to 40 years	350 (49.4%)	230 (30.3%)
41 to 47 years	18 (2.6%)	9 (1.2%)
Past record of miscarriages		
No	618 (87.3%)	728 (96.2%)
Yes	90 (12.6%)	29 (3.8%)
Gestational duration at prenatal appointment		
First trimester	368 (51.9%)	337 (44.4%)
Second trimester	134 (18.9%)	264 (34.8%)
Third trimester	206 (29.1%)	158 (20.8%)
Hypothyroidism		N/A
Prior to pregnancy	611 (86.3%)	
During pregnancy	70 (9.9%)	
Unknown status	27 (3.8%)	
Comorbidities		N/A
Pre-gestational diabetes mellitus	55 (7.8%)	
Gestational diabetes mellitus	150 (21.2%)	
Gestational hypertension	75 (10.6%)	
Chronic hypertension	34 (4.8%)	

 Within both groups, the majority of the women visited during the first trimester, constituting 53.8% in the hypothyroid and 44.4% in the healthy cohort. Furthermore, 86.3% of the hypothyroid women had already been diagnosed with hypothyroidism before conception. The etiology of thyroid disorders was not mentioned in 70.5% cases; however, 26% were diagnosed with Hashimoto’s thyroiditis. The primary three comorbidities prevalent among hypothyroid pregnant women comprised of GDM (21.2%), GH (10.6%), and diabetes mellitus before conception (7.8%).

###  Pregnancy Loss

 There were four subgroups of pregnancy loss in the hypothyroid group. Abortions accounted for more than half of the adverse outcomes (65.7%, n = 46). Stillbirth or IUD and termination of pregnancy were found in 12.9% (n = 9) cases respectively. Finally, there were 8.6% (n = 6) cases of ectopic pregnancy. All cases of pregnancy loss in control healthy women were abortions (100%, n = 108).

###  Maternal Factors Affecting Outcomes

 In the univariate logistic regression analysis, each independent variable such as age of the pregnant women, gestational duration at prenatal appointment, GDM, hypertension, hypothyroidism, and past record of miscarriages were significantly associated with the gestational outcome (Table 2).

**Table 2:** Univariate Analysis of Independent Maternal Factors Affecting Gestational Outcomes in Hypothyroid and Healthy Women

**Maternal Factors**	**Odds Ratio (95% CIs)**	***P *** **Value (Cut-off of≤0.25)**
Age of the mother		< 0.01
19 to 30 years (Ref.)	1.00	
31 to 40 years	1.36 (0.98 – 1.88)	
41 to 47 years	4.47 (1.93 – 0.32)	
Gestational duration at prenatal appointment		< 0.01
First trimester (Ref.)	1.00	
Second trimester	0.45 (0.30 – 0.66)	
Third trimester	0.17 (0.09 – 0.32)	
Past record of miscarriages		< 0.01
Yes	2.93 (1.87 – 4.58)	
No (Ref.)	1.00	
Gestational diabetes mellitus		< 0.01
Yes	0.04 (0.005 – 0.30)	
No (Ref.)	1.00	
Hypothyroidism		0.01
No	1	
Yes	0.68 (0.49 – 0.93)	
Hypertension		0.01
Yes	0.39 (0.17 – 0.91)	
No (Ref.)	1.00	

 The above independent variables were significantly associated with gestational outcome, except hypertension (Table 3) on multivariable logistic regression modelling. In accordance with the findings, women within the age range of 31 to 40 years demonstrated an odds ratio of 1.55 (95% confidence interval, 1.09–2.19) for pregnancy loss, in contrast to those between 19 to 30 years of age. Likewise, women with a past record of miscarriage had an odds ratio of 3.11 (95% confidence interval, 1.90–5.09) for pregnancy loss. This study found that hypothyroid pregnant women had 36% lower odds of suffering from a pregnancy loss. Additionally, mothers with GDM demonstrated 96% lower odds of pregnancy loss compared to those without GDM. Furthermore, pregnant women presenting in their third trimesters exhibited 81% lower odds of pregnancy loss in comparison to those presenting in their first trimester.

**Table 3:** Multivariable Analysis of Maternal Factors Affecting Gestational Outcomes in Hypothyroid and Healthy Women

**Maternal Factors**	**Odds Ratio (95% CIs)**	***P *** **Value (Cut-off of≤0.05)**^a^
Age of the mother		< 0.01
19 to 30 years (Ref.)	1.00	
31 to 40 years	1.55 (1.09 – 2.19)	
41 to 47 years	4.87 (1.92 – 12.28)	
Gestational duration at prenatal appointment		< 0.01
First trimester (Ref.)	1.00	
Second trimester	0.42 (0.28-0.63)	
Third trimester	0.19 (0.10-0.36)	
Past record of miscarriages		
Yes	3.11 (1.90 – 5.09)	0.01
No (Ref.)	1.00	
Gestational diabetes mellitus		< 0.01
Yes	0.04 (0.06 – 0.32)	
No (Ref.)	1.00	
Hypothyroidism		0.01
No	1.00	
Yes	0.62 (0.43-0.89)	

^a^ < 0.01 (Overall model’s *P* value).

###  Comparative Analysis of Hypothyroidism Diagnosed Before or During Gestation

 Before conception, approximately 86.3% of the hypothyroid women had been diagnosed with hypothyroidism (overt as well as subclinical). Pregnancy loss was not statistically significant when hypothyroidism was diagnosed during gestation compared to the diagnosis made before conception (*P* value = 0.628).

###  Effect of Overt Versus Subclinical Categories of Hypothyroidism on Gestational Outcomes

 The type of hypothyroidism in pregnant women was used to classify independent maternal variables (Table 4). Table 5 presents the results of analysis focusing on women with overt and subclinical hypothyroidism during gestation. No substantial difference was observed in factors influencing gestational outcomes between these women categorically. There was no evidence of GDM in cases of overt hypothyroidism. Only in subclinical hypothyroid patients did GDM have a significant effect on the gestational outcome (*P* value = 0.002).

**Table 4:** Independent Maternal Factors Based on Type of Hypothyroidism.

**Maternal Factors**	**Overt Hypothyroidism** **(n=99)** **No. (%)**	**Subclinical Hypothyroidism** **(n=260)** **No. (%)**
Age of the mother		
19 to 30 years	44 (44.4%)	138 (53.1%)
31 to 40 years	52 (52.5%)	113 (43.2%)
41 to 47 years	3 (3.1%)	9 (3.5%)
Past record of miscarriages		
Yes	17 (17.2%)	30 (11.5%)
No	82 (82.8%)	230 (88.5%)
Gestational duration at prenatal appointment		
First trimester	55 (55.6%)	151 (58.1%)
Second trimester	24 (24.2%)	45 (17.3%)
Third trimester	20 (20.2%)	64 (24.6%)
Pre-gestational diabetes mellitus		
Yes	6 (6.1%)	28 (10.8%)
No	93 (93.9%)	231 (89.2%)
Gestational diabetes mellitus		
Yes	11 (11.1%)	52 (20.0%)
No	88 (88.9%)	208 (80%)
Hypertension		
Yes	10 (10.1%)	216 (83.1%)
No	89 (89.9%)	44 (16.9%)

**Table 5:** Subgroup Analysis of Maternal Factors Affecting Gestational Outcomes Based on Type of Hypothyroidism (Either Before or During Pregnancy)

**Maternal Factors**	**Odds Ratio (95% CIs)**	***P *** **Value (Cut-off of≤0.05)**^a^
Overt hypothyroidism (either before or during pregnancy)		0.936
No	1.00	
Yes	0.97(0.53-1.77)	
Age of the mother		0.034
19 to 30 years (Ref.)	1.00	
31 to 40 years	1.57 (1.03 – 2.38)	
41 to 47 years	3.21 (0.83 – 12.39)	
Past record of miscarriages		0.001
Yes	4.32 (2.21 – 8.46)	
No (Ref.)	1.00	
Gestational duration at prenatal appointment		0.001
First trimester (Ref.)	1.00	
Second trimester	0.35 (0.22-0.56)	
Third trimester	0.07 (0.02-0.20)	
Sub clinical hypothyroidism (either before or during pregnancy)		0.06
No	1.00	
Yes	0.63 (0.38-1.02)	
Age of the mother		< 0.01
19 to 30 years (Ref.)	1.00	
31 to 40 years	1.72 (1.16 – 2.55)	
41 to 47 years	4.61 (1.51 – 14.03)	
Past record of miscarriages		< 0.01
Yes	3.87 (2.09 – 7.18)	
No (Ref.)	1.00	
Gestational duration at prenatal appointment		< 0.01
First trimester (Ref.)	1.00	
Second trimester	0.39 (0.24-0.61)	
Third trimester	0.16 (0.07-0.34)	
Gestational diabetes mellitus		< 0.01
Yes	0.11 (0.01 – 0.88)	
No (Ref.)	1.00	

^a^ < 0.05 (Overall model’s *P* value).

## Discussion

 In this study, gestational diabetes and hypothyroidism were found to have a beneficial effect, while the age of the mother, gestational duration at prenatal appointment, and past record of miscarriages were identified to be primary maternal factors influencing gestational outcomes in hypothyroid women. Maternal characteristics and outcomes of the hypothyroid pregnant women have been previously described in a separate study (MHPO-1).^18^

 From a regional perspective, an Indian study showed increased age ( > 30 years) to be associated with miscarriage risk.^19^ Hence, an older pregnant woman with hypothyroidism faces an elevated risk of poor obstetric outcomes compared to a younger counterpart. Our study is also consistent with this outcome.

 There is sufficient statistical information on the link between repeated miscarriages and hypothyroidism.^4^ However, past record of miscarriages as an independent variable contributing to the risk of pregnancy loss in hypothyroid women is not well studied. In a retrospective study, the chances of successive pregnancies were not different between borderline hypothyroid and euthyroid women (55.4% vs 51.3%), despite the fact that the pregnancy loss rate (22 weeks of gestation) was greater in the borderline-subclinical category than in the euthyroid women (29.0% vs 17.9%; *P* value = 0.16).^20^ Different studies have reported adverse fetal outcomes in hypothyroid pregnancies,^9^ whereas some studies describe no significant effect.^21^ However, this study found that the diagnosis of hypothyroidism, in fact, had beneficial effect. TSH levels stayed near euthyroid range in the hypothyroid group and the majority of cases received replacement therapy on timely schedule.

 A recent meta-analysis has shown that subclinical hypothyroidism with positive anti-thyroid antibodies significantly increases the GDM risk (OR 3.22, 95% CI, 1.72-6.03).^22^ In another study, the incidence of thyroid dysfunction was found similar between GDM and non-GDM women.^23^ The existence of both endocrine disorders has a higher effect on adverse pregnancy-related outcomes compared to either comorbidity alone.^24^ Furthermore, existing studies have primarily focused on the occurrence of GDM in pregnancies with hypothyroidism,^25^ as opposed to assessing diabetes as an independent factor influencing gestational outcomes in women with hypothyroidism, as explored in this study. Our data revealed a noteworthy protective effect on gestational outcomes among hypothyroid women (specifically subclinical cases) with GDM. We speculate that this phenomenon can be attributed to the existence of a specialized healthcare system already recognized for GDM patients in our hospital, featuring a combined clinic equipped with experts from different specialties dedicated to addressing this type of patients.

 A study from Bangladesh failed to identify a statistically significant relationship between first-trimester pregnancy loss and elevated TSH levels.^26^ Most of the patients were diagnosed as hypothyroid before conception in this study, and were undergoing levothyroxine replacement therapy. Despite the majority not having preconception TSH levels measured, those presenting around the second trimester were more prone to experiencing loss of pregnancy compared to women presenting in the third trimester.

 The retrospective study design limited our ability to comprehensively compare all maternal factors between hypothyroid and control groups. Besides, there was a high prevalence of pre-pregnancy (overt and subclinical) hypothyroidism in the study, which can represent a selection bias with respect to gestational outcomes. In addition, due to the retrospective design of this study, there is an inherited unmeasured confounding of independent variables associated with the gestational outcomes of women with hypothyroidism. Nevertheless, to our knowledge, this study represents the first of its kind in Pakistan, with an adequate sample population for the examination and interpretation of findings. We advocate for future research employing prospective and/or case-control study designs to further validate the reported outcomes.

## Conclusion

 In this study, the age of the mother, gestational duration at prenatal appointment, and a past record of miscarriages were identified as significant factors influencing adverse gestational outcomes. Interestingly, gestational diabetes and hypothyroidism appeared to exhibit a protective effect. It is recommended that women of reproductive age should receive health education to encourage timely visit to the obstetricians and/or endocrinologists to prevent against adverse gestational outcomes.
